# Pathway-Based Genome-Wide Association Studies for Two Meat Production Traits in Simmental Cattle

**DOI:** 10.1038/srep18389

**Published:** 2015-12-17

**Authors:** Huizhong Fan, Yang Wu, Xiaojing Zhou, Jiangwei Xia, Wengang Zhang, Yuxin Song, Fei Liu, Yan Chen, Lupei Zhang, Xue Gao, Huijiang Gao, Junya Li

**Affiliations:** 1 Institute of Animal Science, Chinese Academy of Agricultural Science, Beijing 100193, China; 2 Department of Mathematics, Heilongjiang Bayi Agricultural University, 163319 Daqing, China; 3 College of Animal Science and Technology, Agricultural University of Hebei Province, Baoding, 071001, China

## Abstract

Most single nucleotide polymorphisms (SNPs) detected by genome-wide association studies (GWAS), explain only a small fraction of phenotypic variation. Pathway-based GWAS were proposed to improve the proportion of genes for some human complex traits that could be explained by enriching a mass of SNPs within genetic groups. However, few attempts have been made to describe the quantitative traits in domestic animals. In this study, we used a dataset with approximately 7,700,000 SNPs from 807 Simmental cattle and analyzed live weight and longissimus muscle area using a modified pathway-based GWAS method to orthogonalise the highly linked SNPs within each gene using principal component analysis (PCA). As a result, of the 262 biological pathways of cattle collected from the KEGG database, the gamma aminobutyric acid (GABA)ergic synapse pathway and the non-alcoholic fatty liver disease (NAFLD) pathway were significantly associated with the two traits analyzed. The GABAergic synapse pathway was biologically applicable to the traits analyzed because of its roles in feed intake and weight gain. The proposed method had high statistical power and a low false discovery rate, compared to those of the smallest P-value and SNP set enrichment analysis methods.

Genome-wide association studies (GWAS) have become a powerful and increasingly affordable tool to discover the genetic bases of complex diseases in humans^1^,^2^,^3^ and economically important traits in domestic animals after development of genome sequencing and high throughput single nucleotide polymorphism (SNP) genotyping technologies^4^,^5^,^6^,^7^,^8^,^9^. Numerous GWAS studies have been performed in livestock and many novel genes associated with economically important traits have been detected^10^,^11^. However, these data are always analyzed considering the SNPs independently and testing the alleles at each locus for an association^12^. Thus, the most significant SNP or neighboring genes are the focus and little attention is given to the remainder^13^. However, this approach has some limitations. First, the SNPs may not meet the threshold for statistical significance due to strict criteria after adjusting for multiple testing^14^. Alternatively, significant SNPs may be located in genomic regions without any unifying biological theme. Moreover, complex quantitative traits are usually determined by many genes with small effects; thus, genetic variants that may have significant combined genetic effects but make only a small individual contribution may be missed by a single-SNP analysis^15^.

Numerous strategies and statistical approaches have been developed to meet the conceptual and technical challenges and take full advantage of the wide opportunities provided by GWAS^16^,^17^. One such approach is a pathway-based analysis, which considers cumulative associations between the outcome and a group of SNPs or genes in a biological pathway and greatly complements the SNP/gene approach to understand the genetic reasons for complex traits^18^,^19^,^20^,^21^,^22^. Pathway-based analyses are used to investigate how a group of genetic variants in the same biological pathway are associated with quantitative traits, which can help holistically unravel the complex genetic structure of phenotypic variations. Moreover, this approach substantially reduces the multiple testing burden after genes are grouped into pathways for association testing and biological knowledge is incorporated into the analysis^23^. Several pathway-based GWAS algorithms have been developed and implemented in different software packages^20^,^24^,^25^,^26^,^27^,^28^,^29^. One of the most popular pathway-based algorithms is the smallest P-value method, which uses the SNP with the strongest association to represent a gene^20^. However, choosing the smallest P-value to represent a gene might not be optimal in situations when multiple SNPs explain more variance than the single most significant SNP. Moreover, this approach favors larger genes, as larger genes may have a higher chance of containing significant SNPs^29^. Another popular pathway-based GWAS algorithm was proposed by Holden *et al.*^24^. This method uses all available SNPs contained in a gene to represent the gene. However, this method is computationally insensitive and may not be applicable for GWAS with millions of SNPs. Weng *et al.*^29^ developed a new SNP-based analysis called SNP Set Enrichment Analysis (SSEA), which selects several different SNPs to represent each gene using an adaptive truncated product statistic, which effectively solves the problem of determining the number of SNPs and selecting the best SNP for each gene. However, this strategy is based on the assumption that the P-values of the SNPs in genes are independent but they are actually in linkage disequilibrium.

Some GWAS studies have been performed in Korean Hanwoo cattle^30^, Korean beef cattle^31^, and Australian taurine and indicine cattle^4^ to detect SNPs associated with carcass and meat quantity traits. However, none of these reports focused on pathways in beef cattle. In this study, we propose a modified pathway-based GWAS method that calculates gene-phenotype statistics using the independent principal components (PC) of multiple SNPs within a gene and then uses the Kolmogorov–Smirnov statistic to infer the genetic association between each pathway and trait of interest. A total of 7,700,000 SNPs were genotyped in 807 Simmental cattle to detect pathways for live weight (LW) and longissimus muscle area (LMA); 262 biological pathways for cattle were collected from the KEGG database.

## Materials and Methods

### Ethics statement

All animal procedures were in strict accordance with the guidelines proposed by the Chinese Council on Animal Care, and all protocols were approved by the Science Research Department of the Institute of Animal Science, Chinese Academy of Agricultural Sciences (Beijing, China). The use of animals and private land in this study was approved by the respective owners.

### Animal resource and phenotypes

As part of our resource population of Simmental cattle established in Ulgai, Xilingol league, Inner Mongolia, China, the mapping population consisted of 814 young Simmental bulls born in 2009–2012. After weaning, the cattle were moved to the Beijing Jinweifuren Cattle Farm for feedlot finishing under the same feeding and management system. All bulls were observed for growth and developmental traits until slaughter at 16–18 months of age. This study focuses on the phenotypic traits associated with cattle meat production, so carcass and meat traits were measured according to the Institutional Meat Purchase Specifications for fresh beef guidelines during the slaughter period. Among them, LW and LMA were chosen for the pathway-based GWAS analysis. LW was measured before slaughter after fasting for 24 hours, and LMA was measured at the interface of ribs 12 and 13 48 hours postmortem using a grid expressed in square centimeters. Evaluators counted the number of dots on the grid that were over the muscle area. Each dot was equal to 1 cm^2^. Snowdragon cattle crossed with Japanese Black cattle and a local breed were used to validate our GWAS findings. This replicate sample consisted of 451 Snowdragon cattle from seven farms in Liaoning Province, China. The cattle were fattened at the Snowdragon Beef Limited Company. Both LW and LMA were measured, as in the Simmental sample.

### Sample genotyping and quality control

Blood samples were collected during the regular farm quarantine inspection. Genomic DNA was extracted from blood using the TIANamp Blood DNA Kit (Tiangen Biotech Co., Ltd., Beijing, China). DNAs with an A260/280 ratio of 1.8–2.0 were subjected to further analysis. The Illumina BovineHD BeadChip (Illumina Inc., San Diego, CA USA; http://www.illumina.com/documents/products/datasheets/datasheet_bovineHD.pdf) with 774,660 SNPs was chosen for individual genotyping. Details of BovineHD BeadChip can be seen. The SNPs were uniformly distributed on the whole bovine genome with a mean inter-marker space of 3.43 kb. The genotyping platform adopted in this study was Illumina’s Infinium II Assay. Samples were genotyped using Illumina BEADSTUDIO ver. 2009, and SNP chips were scanned and analyzed using Infinium GenomeStudio software.

PLINK software (v1.9, http://pngu.mgh.harvard.edu/~purcell/plink/) was used to exclude individuals and remove SNPs for quality control. The quality control procedure was as follows: individuals with >10% missing genotypes or a Mendelian SNP genotype error >2% were excluded. SNPs with call rates <90%, minor allele frequencies (MAF) <5%, <5 genotype appearances, or Hardy–Weinberg equilibrium (HWE) <10^−6^ were excluded. All misplaced SNPs were excluded from the analysis.

### Identifying pathways

We retrieved all cattle pathways from the KEGG^32^ pathway database (http://www.genome.jp/kegg/) to identify pathways that potentially contribute to meat production traits in cattle and selected 280 annotated pathways for analysis. Each gene was covered by at least five SNPs in our genome dataset to relieve multiple testing issues and to avoid testing for narrow functional pathways. Pathways with fewer than five or more than 300 genes were filtered.

All genes (including coding, small non-coding genes, and pseudogenes) involved in the pathways were based on the *Bos taurus* UMD 3.1 sequence, and all assemblies were obtained from the Ensembl Genes 80 Database at BioMart (http://asia.ensembl.org/biomart/martview). In addition, if genes were involved in two pathways, both were included in the analysis.

### Phenotypic correction

After collecting the original data, the phenotypes were corrected in advance for fixed effects, including year, season, fattening days, and entering weight using the following equation:


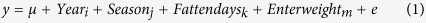


where y is the phenotypic value, *μ* is the population mean, Year_i_ is slaughtering year, divided into three groups (2009, 2010 and 2011); Season_j_ is the calving season, including three levels (November–April, May–August, and September–October). Fattendays_k_ and Enterweight are continuous variables. Fattendays_k_ is the number of days since entering the fattening farm to slaughter, and Enterweight_m_ is live weight upon entering the fattening farm. e is a random residual for the subsequent association study with the SNPs.

### PC construction within a gene

1. *Eigenvalue calculation*: Eigenvalues and eigenvectors were calculated using the covariance matrix for SNP genotype indictors.
2. *Principal component selection*: PCs were selected based on a cumulative contributed proportion >85% for the ranked eigenvalue.
3. *PC construction*: PCs were calculated by multiplying the eigenvectors corresponding to the selected eigenvalues by the SNP genotype indictor matrix within each gene.

### Gene-phenotype statistical calculations

The PCs were mutually independent within a gene, so the multiple regression analysis for the corrected phenotypes on the PCs was changed to a simple regression using the PC. A simple regression analysis is equivalent to a correlation analysis between corrected phenotypes and PCs, so the correlation coefficient between two variables was used to calculate the P-value of the PC. In addition, if the population in the mapping population structure was stratified, the regression model was further corrected using PCs, as coverable from a portion of the bovine genome SNPs. The model was:


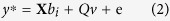


where y* is the corrected phenotype, **b**_**i**_ is the regression coefficient of the phenotype on the PCs, **X** is the PC, **v** is the effect of population structure, **Q** is the corresponding population structure matrix constructed using the first three PCs from the portion of bovine genome SNPs, and **e** is the vector of residual errors with **e~N (0, I**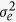).

The maxmean statistical strategy developed by Efron *et al.*^33^ was applied to calculate the gene-phenotype statistical value for a specific gene, as follows:


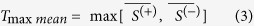


where 
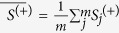
 represents the positive mean of the PC-phenotype association value and 
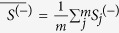
 is the negative mean of the PC-phenotype value.

### Pathway-based enrichment analysis

1. *Ranking statistical values for the gene-phenotype associations*: All genes were ranked by sorting their gene-phenotype statistical values from largest to smallest in a gene list (r_1_ , r_2_ … r_N_ ).
2. *Calculation of enrichment score*: The enrichment score (ES) value is a weighted Kolmogorov–Smirnov^14^,^34^ statistical value that reflects overrepresentation of a given pathway S. The score is calculated as:
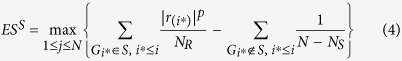
where 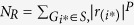, and p is a parameter that gives weight to genes with extreme statistical values. Here, p was 1.
3. *Phenotype permutation and significance assessment*: Permutation procedures were used to estimate the ES significance level. In each permutation, we first shuffled the phenotype labels and repeated the previous two steps to calculate ES for each pathway (ES_null_). Due to the large size of the dataset, computational complexity was extremely high when the number of permutations was large. Thus, 1,000 permutation-cycles were used to generate the permutated datasets. The significance of an observed ES^s^ for a pathway (nominal p-value) was estimated as the percentage of permutations whose ES^s^_null_ values were greater than the observed ES^s^.
4. *Multiple-testing adjustments*: Multiple-testing adjustments were used to compare pathways with different numbers of genes. A normalized ES (NES) was constructed based on the observed ES and the mean and standard deviation (SD) of ES^s^_null_.

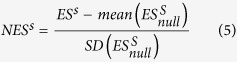


Then, false discovery rate (FDR) was used to adjust for multiple-hypothesis testing to obtain more reliable results. FDR maintains the fraction of expected false-positive findings below a certain threshold. For a given pathway, let *NES*^s*^_observe_ denote the NES in the observed data. The FDR q-value was calculated as the ratio of the fraction of all permutations with 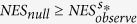 to the fraction of observed pathways with 
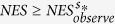
.


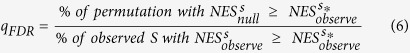


After correcting for multiple-testing, the significance criteria for a pathway are that the q_FDR_ and nominal p-values are <0.05 and 0.01, respectively.

## Results

### SNP quality results and phenotype statistics

According to the quality control criteria, seven individuals had >10% missing genotypes. Additionally, 59,621, 14,366, and 9,142 SNPs with call rates <90%, MAF <5%, and HWE <10^−6^ were excluded. As a result, 691,531 SNPs were used in the pathway-based GWAS analysis. A total of 262 pathways were collected for cattle, which covered 95,672 SNPs, so 595,859 misplaced SNPs were excluded from the pathway analysis. The means, SDs, standard errors, and ranges for the LW and LMA traits are presented in Table 1.

### Population stratification assessment

**Figure 1:**
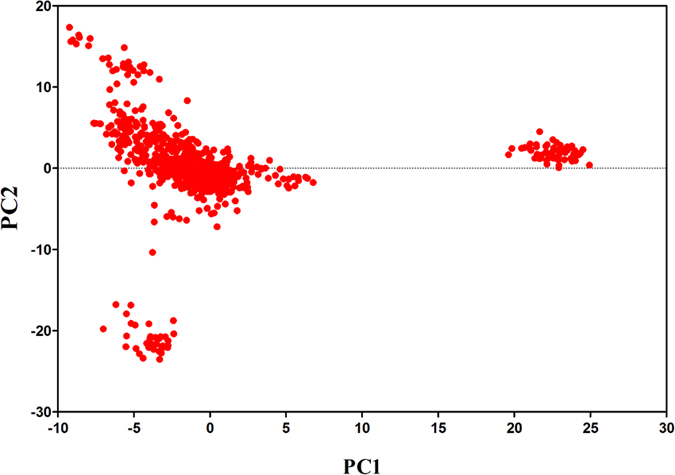
Population structure map drawn from the first two principal components.

The population structure was constructed using a portion of the SNPs by clustering with PCA. As illustrated in Fig. 1, the population structure was drawn based on PC1 and PC2. The three major sectors indicated that the sample population was stratified; thus, population stratification was corrected for in the analysis. Population stratification may have occurred because the experimental cattle came from different farms and had different genetic backgrounds.

### Pathway-based association analysis

**Figure 2:**
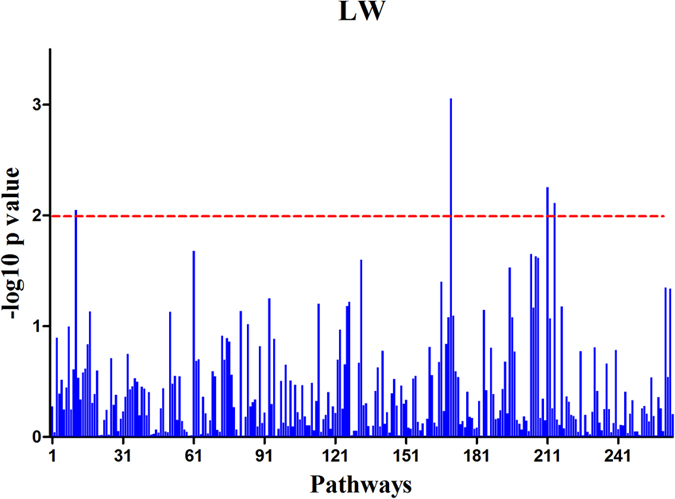
Log10 (P-value) values of all 262 pathways for the live weight trait.

We discovered four pathways for LW with normal P-values ≤ 0.01, but the FDR q-value was >0.05. The results of these pathways for LW are listed in Table 2. Of the four pathways, the gamma aminobutyric acid (GABA)ergic synapse pathway had the highest ES score of 0.40 with a nominal P-value = 0.000876, showing the strongest evidence of association with LW, as illustrated in Fig. 2. The pathway involved 87 genes reported by the KEGG database but only 62 genes were covered by our SNP set. Among these 62 genes, seven ranked among the top 1,000 genes in the gene list, and the *GNG11* gene was the most significant. Genetic variance in the pathway was calculated using the cumulative genetic variance for all PCs of the genes in the pathway and a multiple linear model to estimate heritability of the pathways. The four pathways had heritability values of 0.04, 0.05, 0.07, and 0.06 for GABAergic synapse, morphine addiction, bacterial invasion of epithelial cells, and synthesis and degradation of ketone bodies, respectively. These four pathways included 0.22 of total phenotypic and genetic variation.

**Figure 3:**
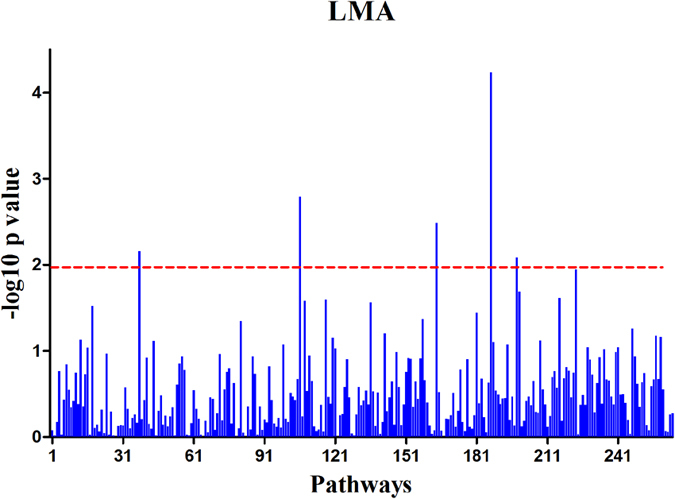
Log10 (P-value) values of all 262 pathways for the longissimus muscle area trait.

As shown in Table 3, five LMA pathways were statistically inferred to be significant at a P-value of 0.01. Among these pathways, only the non-alcoholic fatty liver disease (NAFLD) pathway met the nominal P-value and q_FDR_ criteria and presented the most distinct association with LMA (Fig. 3). There were 15 leading-edge genes within the NAFLD pathway, including *NDUFA6, IRS1, NDUFAB1, IL6R, SREBF1, ADIPOR2, PRKAA2, NDUFA3, NR1H3, NDUFS5, UQCR11, PIK3R3, RELA, COX4I2,* and *NDUFA4*. The five pathways identified for LMA explained phenotypic variation of 0.05, 0.07, 0.10, 0.09, and 0.11 for NAFLD, cytokine-cytokine receptor interactions, the synaptic vesicle cycle, other glycan degradation, and carbohydrate digestion and absorption, respectively. These five pathways included 0.42 of the total phenotypic and genetic variation.

**Figure 4:**
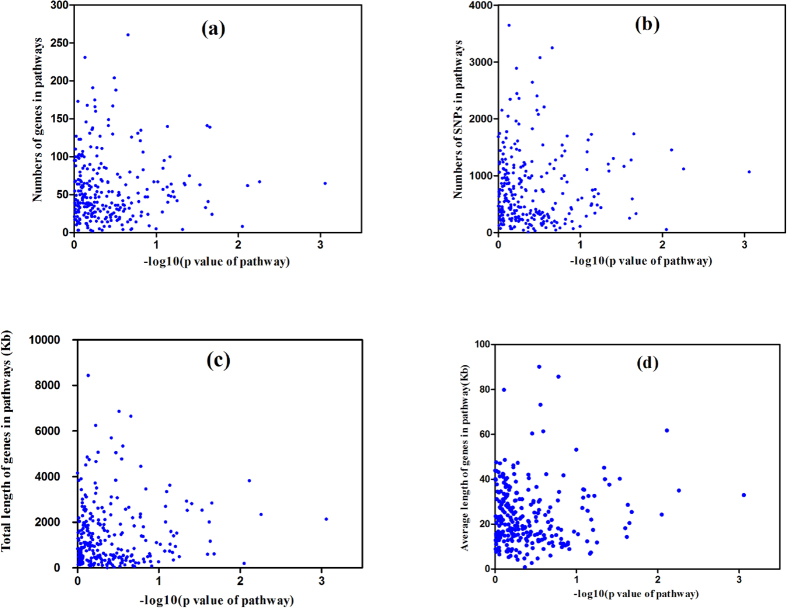
Significance of the pathway (–log10 and P-values) for the live weight trait versus (**a**) the number of genes in the pathways, (**b**) the number of significant single nucleotide polymorphisms (SNPs) in the pathways, (**c**) total length (kb) of genes in the pathways, and (**d**) mean length (kb) of the genes in the pathways.

Furthermore, we prepared plots of pathway size, gene size, total gene bp content, and mean gene content against the –LogP values of the selected pathways to investigate the effects of potential factors on detecting the pathways using our method. The associations between these factors and the significance of the pathways confirmed the capability of the maxmean strategy and permutations to reduce bias caused by the gene and pathway sizes (Fig. 4).

### Comparison with other methods

We also applied other methods, such as the smallest P-value^20^ and the secondary structure element alignment (SSEA) methods^29^, to analyze our GWAS dataset. As a result, the smallest P-value method only detected the GABAergic synapse pathway for LW and the NAFLD pathway for LMA based on the nominal P-value. Furthermore, the two identified pathways were statistically inferred to be not significant according to the FDR value. The SSEA method detected three of the pathways identified by our method, but these pathways had high nominal P- and FDR q-values (see Supplemental Table S2). This result suggests that our method improved the power of detecting the pathways. Additionally, 17 and 20 leading-edge genes for the NAFLD pathway and LMA were detected using the smallest P-value and SSEA methods, respectively. Among these leading-edge genes, seven and nine genes detected by the smallest P-value and SSEA methods overlapped with our method.

### Validation of the pathways identified

We carried out a replication analysis with a Snowdragon cattle sample to further test the associations using our method and to confirm our GWAS findings in the discovery cohort. A total of 262 pathways were analyzed, as in the discovery cohort. This analysis detected three pathways significantly associated with the LW trait. The observed NES was 2.54, the observed ES was 0.32, the nominal P-value was 0.005, and the FDR q-value was 0.30 for the GABAergic synapse pathway, based on 1,000 permutations. This result indicates that the GABAergic synapse pathway was associated with LW. In addition, applying our method to the LMA trait resulted in the discovery of four pathways with nominal P-values <0.01. The NAFLD pathway, with an observed ES value of 0.29, a MES value of 3.31, a P-value of 0.0001, and a FDR q-value of 0.04 was most significantly associated with LMA.

## Discussion

We developed a modified pathway-based GWAS analysis method, where maxmean statistics were calculated for each gene using independent PCs from multiple SNPs within a gene. We found that the GABAergic synapse and NAFLD pathways were significantly associated with LW and LMA, respectively, using approximately 7,700,000 SNPs in 807 Simmental cattle. Of them, the GABAergic synapse pathway was associated with animal feed intake and weight gain. Our method detected the pathways with high statistical power and low FDR and identified the same pathways detected by the smallest P-value and SSEA methods.

Different from common GWAS where SNPs are the genetic units analyzed, the GWAS conducted here identified the pathways regulating quantitative traits. The biologically meaningful pathways detected were useful to interpret the GWAS results and included different effects of the significant and non-significant SNPs from a common GWAS. Compared to previous pathway-based GWAS strategies, our modified method orthogonalised the SNPs within each gene using PCA to make the highly linked SNPs independent, which helped formulate the statistics for a gene using multiple linked SNPs. Of course, the PCAs to formulate the gene statistics were chosen using as much of the SNP information as possible. This approach differs from simply removing the associated SNPs. Generally, quantitative trait loci (QTL) for a trait with low heritability are difficult to detect because QTL heritability is also low, as it is controlled by multiple genes. In addition, our proposed method is suitable for traits controlled by rare alleles, as the PCA contained all SNPs within each gene.

A large number of GWAS have been performed for pathways since Wang *et al.*^20^ initially proposed the pathway-based GWAS approach. Based on 37,000 SNPs across the genome of 618 unrelated elder Han Chinese, Pan *et al.*^35^ reported that the regulation of autophagy (ROA) pathway of 626 analyzed biological pathways is associated with human stature. Zhang *et al.*^36^ identified the most significant ROA pathway for the bone mineral density (BMD) trait by analyzing approximately 500,000 SNPs from 963 biological pathways/gene sets of 984 unrelated Caucasians. Additionally, the glutamate receptor pathway is identified by Wang *et al.*^20^ by analyzing the GWAS dataset on Parkinson’s disease of Fung *et al.*^37^. Attempts have been made to assess quantitative traits of litters in domestic animals, but only the RNASE5 pathway was associated with the milk yield trait in dairy cattle^38^. In this study, we examined approximately 770,000 SNPs from 807 Simmental cattle and analyzed 262 pathways from the KEGG database. We found that GABAergic synapse and NAFLD pathways were significantly associated with the LW and LMA meat production traits.

GABA is a neurotransmitter widely distributed in the central nervous system. GABA is synthesized from glutamate through decarboxylation^39^ and plays an important role regulating feeding behavior in the hypothalamus. Seane *et al.*^40^ reported that injecting 160 nmol muscimol (GABA-A receptor agonist) into the lateral ventricle increases feed intake of satiated sheep, suggesting that neuronal sensitivity to GABA is related to the control of feeding behavior in ruminant animals. Stratford *et al.*^41^ reported that injecting muscimol and baclofen (GABA-B receptor agonist) into the nucleus accumbens centrum increases feed intake in engorged rats. Additionally, Fan *et al.*^42^ showed that feeding GABA increases feed intake and weight gain in growing pigs, whereas Wang *et al.*^43^ demonstrated that adding rumen-protected GABA was beneficial to early lactation in dairy cows in terms of feed intake, lactation performance, and health. In this study, we found that GABA was significantly associated with the LW trait in Simmental cattle. We hypothesized that dietary GABA supplementation would increase feed intake and LW gain in beef cattle; however, this hypothesis requires further experimentation.

A critical component for a successful pathway-based analysis is the ability to identify competitive pathways related to the trait. The pathways available in livestock animals remain very limited, as most publically released gene sets were generated from humans. As a consequence, the pathways discovered here are likely to be incomplete. Our method will perform better when more domestic animal pathways become available, at that time, more significant pathways related to meat production traits will be detected.

## Additional Information

**How to cite this article**: Fan, H. *et al.* Pathway-Based Genome-Wide Association Studies for Two Meat Production Traits in Simmental Cattle. *Sci. Rep.* **5**, 18389; doi: 10.1038/srep18389 (2015).

## Supplementary Material

Supplementary Information

Supplementary Dataset 1

## Figures and Tables

**Table 1:** Descriptive statistics for the two Simmental cattle production traits.

Traits	Mean	Standard deviation	Standard error	Maximum	Minimum
LW	491.64 kg	57.53 kg	2.03 kg	711 kg	318 kg
LMA	82.85 cm^2^	12.23 cm^2^	0.43 cm^2^	150 cm^2^	51 cm^2^

LW, live weight; LMA, longissimus muscle area.

**Table 2:** Four significant pathways identified for the live weight (LW) trait.

Pathways	Description	ES	NES	P value	FDR	Heritabilities
bta04727	GABAergic synapse	0.40	3.06	8.76E-04	0.24	0.04
bta05032	Morphine addiction	0.38	2.51	5.53E-03	0.76	0.05
bta05100	Bacterial invasion of epithelial cells	0.39	2.39	7.73E-03	0.71	0.07
bta00072	Synthesis and degradation of ketone bodies	0.70	2.34	8.96E-03	0.61	0.06

ES, enrichment score; NES, normalized enrichment score; FDR, false discovery rate.

**Table 3:** Five significant pathways (P < 0.01) identified for the longissimus muscle area (LMA) trait.

Pathways		ES	NES	P value	FDR	Heritabilities
bta04932	Non-alcoholic fatty liver disease (NAFLD)	0.38	3.73	5.840E-05	0.02	0.05
bta04060	Cytokine-cytokine receptor interaction	0.33	2.90	1.613E-03	0.22	0.07
bta04721	Synaptic vesicle cycle	0.38	2.68	3.263E-03	0.30	0.10
bta00511	Other glycan degradation	0.53	2.43	6.923E-03	0.47	0.09
bta04973	Carbohydrate digestion and absorption	0.50	2.37	8.234E-03	0.45	0.11

ES, enrichment score; NES, normalized enrichment score; FDR, false discovery rate.
