# Supplementary material for: Pathway-Based Genome-Wide Association Studies for Two Meat Production Traits in Simmental Cattle
Source: Sci Rep. 2015 Dec 17;5:18389. doi: 10.1038/srep18389 (PMC4682090; doi:10.1038/srep18389)
Supplement: Supplementary Information [file srep18389-s1.pdf]

Pathway-Based Genome-Wide Association Studies for Two Meat Production Traits in Simmental Cattle

Huizhong Fan[1], Yang Wu[1], Xiaojing Zhou[2], Jiangwei Xia[1], Wengang Zhang[1], Yuxin Song[1], Fei Liu[1,3], Yan Chen[1], Lupei Zhang[1], Xue Gao[1], Huijiang Gao[1*], and Junya Li[1*]

1. Institute of Animal Science, Chinese Academy of Agricultural Science, Beijing 100193, China
2. Department of Mathematics, Heilongjiang Bayi Agricultural University, 163319 Daqing, China
3. College of Animal Science and Technology, Agricultural University of Hebei Province, Baoding, 071001, China

[*]Corresponding author: Huijiang Gao. Email: gaohj111@sina.com

Junya Li. Email: JL1@iascaas.net.cn

Emails of the other authors:

Huizhong Fan: fanhuizhong1990@qq.com Yang Wu: wuyangf7@126.com

Xiaojing Zhou: 964703463@qq.com Jiangwei Xia: 290466673@qq.com

Wengang Zhang: 1015210729@qq.com Yuxin Song: 2361278555@qq.com

Fei Liu: liufeiyjy@163.com Yan Chen: chenyan0204@163.com

Lupei Zhang: lpzhang@iascaas.net.cn Xue Gao: gaoxue76@126.com

Table S2: The result of significant pathways (P<0.01) discovered by Smallest P-value method and SSEA method.

Method	Trait	Pathway	Description	ES	NES	P value	FDR
Smallest P-value method	LW	bta04727	GABAergic synapse	0.35	2.76	1.121E-04	0.26
	LMA	bta04932	Non-alcoholic fatty liver disease (NAFLD)	0.30	3.16	1.837E-04	0.14
SSEA method	LW	bta04727	GABAergic synapse	0.38	2.99	3.061E-04	0.21
	LMA	bta04932	Non-alcoholic fatty liver disease (NAFLD)	0.35	3.47	2.177E-05	0.08
		bta04060	Cytokine-cytokine receptor interaction	0.30	2.53	7.533E-02	0.25

LW, live weight; LMA, longissimus muscle area. ES, enrichment score; NES, normalized enrichment score; FDR, false discovery rate.
